# Development of nomograms for prognostication of patients with primary soft tissue sarcomas of the trunk and extremity: report from the Bone and Soft Tissue Tumor Registry in Japan

**DOI:** 10.1186/s12885-019-5875-y

**Published:** 2019-07-04

**Authors:** Masaya Sekimizu, Koichi Ogura, Hideo Yasunaga, Hiroki Matsui, Sakae Tanaka, Katsunori Inagaki, Akira Kawai

**Affiliations:** 1 Department of Musculoskeletal Oncology, National Cancer Center Hospital, 5-1-1 Tsukiji, Chuo-ku, Tokyo, 104-0045 Japan; 2 Department of Health Economics and Epidemiology Research, School of Public Health, The University of Tokyo, Tokyo, Japan; 3 Department of Orthopaedic Surgery, The University of Tokyo, Tokyo, Japan; 4 Department of Orthopaedic Surgery, Showa University, Tokyo, Japan

**Keywords:** Nomogram, Soft tissue sarcoma, Prognosis, Asian

## Abstract

**Background:**

The use of nomograms for prognostication of individual cancer patients has been recommended in order to facilitate precision medicine. However, models for patients with soft tissue sarcomas (STSs) are limited because of the rarity and heterogeneity of such cancers. In addition, no model has been developed on the basis of an Asian cohort. Here, we attempted to develop and internally validate nomograms for patients with localized STSs of the trunk and extremity.

**Methods:**

This study retrospectively extracted 2827 patients with primary trunk and extremity STSs after definitive surgery using the Bone and Soft Tissue Tumor Registry, which is a nationwide sarcoma database in Japan. We developed three nomograms predicting the probability of local recurrence-free survival (LRFS), distant metastasis-free survival (DMFS) and disease-specific survival (DSS) at 2 years after surgery, using the Cox multivariate model. The nomograms were internally validated for discrimination and calibration using bootstrap resampling and assessed for their clinical applicability by decision curve analysis (DCA).

**Results:**

Local recurrence, distant metastasis and disease-specific death occurred in 241 patients (8.5%), 554 patients (19.6%) and 230 patients (8.1%), respectively. Histological diagnosis, grade and tumor size strongly influenced all three endpoints. The nomograms predicted accurately the probability of LRFS, DMFS and DSS (concordance index: 0.73, 0.70 and 0.75, respectively). DCA demonstrated that our nomograms had clinical applicability.

**Conclusion:**

We have developed the first nomograms for STSs based on an Asian cohort. These nomograms allowed accurate prediction of LRFS, DMFS and DSS at 2 years after definitive surgery, and can be used as a guide by clinicians for appropriate follow-up and counseling of patients.

**Electronic supplementary material:**

The online version of this article (10.1186/s12885-019-5875-y) contains supplementary material, which is available to authorized users.

## Background

Soft tissue sarcomas (STSs) are a heterogeneous group of malignant tumors that can arise anywhere in the body, making their clinical course variable and the development of a meaningful staging system difficult. With this background in mind, the current eighth edition of the American Joint Committee on Cancer (AJCC) staging system has attempted to incorporate the anatomic primary tumor site as one of the prognostic variables in the T stage, as well as tumor size [1]. Some important factors other than the AJCC staging are also reportedly associated with survival in STS patients, including age, histologic diagnosis, surgical margin, and radiotherapy [2–12]. Estimation of accurate clinical course using these factors will enable clinicians to select appropriate patients who can gain benefit from invasive treatment and may improve prognosis for patients. Therefore, in order to achieve precision medicine, more accurate and personalized outcome prediction tools that can incorporate several prognostic factors influencing survival are required [13].

The nomogram, a graphical calculation tool designed to allow prediction of the overall probability of a specific outcome for any individual patient, can provide clinicians with accurate estimates of prognosis for several cancers. Kattan et al. [8] developed the first nomogram for general site STSs based on the 2002 World Health Organization (WHO) classification, and other nomograms for STSs were then subsequently reported, especially for site-specific tumors [10, 12]. However, there are some major concerns related to the practical use of such nomograms: 1) since the general site nomogram proposed by Kattan et al. [8], no practical nomograms for patients with trunk STS based on the latest WHO histological classification (2013) have been developed [8, 14]; and 2) previous nomograms were developed on the basis of Caucasian cohorts in countries where treatment strategies including the extent of the surgical margin and indications for radiotherapy differed from those involving Asian cohorts [8–12]. Furthermore, to our knowledge, there are no reported nomograms which can concurrently predict following three major outcomes during clinical course of STS: local recurrence-free survival (LRFS), distant metastasis-free survival (DMFS), and disease-specific survival (DSS).

Therefore, in the present study, we aimed to develop nomograms for predicting LRFS, DMFS, and DSS in patients undergoing definitive surgery for trunk and extremity STSs by analyzing data from the Bone and Soft Tissue Tumor (BSTT) Registry in Japan, which is a nationwide organ-specific cancer registry for bone and soft tissue tumors [2, 15].

## Methods

### Data source

The BSTT Registry is a nationwide organ-specific cancer registry for bone and soft tissue tumors in Japan. Details of the BSTT have been reported elsewhere [2, 15]. Detailed data for patients treated at the participating hospitals are collected annually in a clinician-oriented manner. The survey collects data in two sets. The first survey is conducted annually in May for patients treated between January 1 and December 31 of the previous year, and includes the following data for each patient: 1) basic data related to the patient: hospital, sex, age, date of diagnosis, status at the first visit, etc.; 2) information on the tumor: origin of the tumor (bone, soft tissue), histologic details (malignant or benign, and diagnosis), tumor location, the data required for TNM and Enneking staging (tumor size, nodal or distant metastasis, and histologic grade for malignant tumors.); 3) information on surgery: date of definitive surgery, type of surgery, reconstruction details, additional surgery for complications, etc.; and 4) information on treatments other than surgery: details of chemotherapy and radiotherapy. The second survey collects information on prognosis at 2, 5, and 10 years after the initial registration only for patients with bone and soft tissue sarcomas. It includes information on several outcome measures at the time of the latest follow-up, such as local recurrence, distant metastasis, oncologic outcome and limb salvage status. Use of the BSTT Registry for the purposes of clinical research was initiated in 2014 after approval from the Musculoskeletal Tumor Committee of the Japanese orthopaedic association (JOA). Approvals for the present study were obtained from the Institutional Review Boards of the JOA and National Cancer Center (2018–085).

### Study design and eligibility

Data were obtained from the BSTT Registry during 2006–2015. The inclusion criteria for patients were as follows: 1) a diagnosis of primary STS with no distant metastasis (N0 M0 or N1 M0) arising from the trunk or extremities, 2) aged 18 years or older at diagnosis, and 3) having undergone definitive surgery with curative intent. We defined STSs of the trunk as tumors arising from the trunk excluding the head, those of the upper extremity as tumors arising from the shoulder girdle to the hand, and those of the lower extremity as tumors arising from the pelvic girdle (excluding endopelvic tumors) to the foot. STSs were defined as malignant soft tissue tumors (both low and high grade) based on the 2013 WHO classification [14]. Patients with benign or intermediate STSs were excluded. Patients with soft tissue Ewing sarcoma and alveolar or embryonal rhabdomyosarcoma were also excluded, in view of the characteristic natural course and treatment strategies for these tumors.

### Prognostic variables

For this study, we extracted the following data: patient sex, age, tumor size, tumor site (trunk, upper extremity, or lower extremity), tumor depth, histological grade and diagnosis, nodal metastasis status (negative or positive) at diagnosis, surgical margin after surgery, and perioperative adjuvant therapy status. Tumor size was the maximum diameter measured by imaging modalities at the time of diagnosis. Histological grade was classified as either low or high grade. Tumor depth was assessed as superficial or deep relative to the investing fascia. Surgical margin status was defined microscopically as either positive (intralesional) or negative (marginal or wide). Histological diagnosis was based on the latest WHO criteria and grouped into 10 categories [14]: myxoid liposarcoma (MLS), leiomyosarcoma (LMS), dedifferentiated liposarcoma (DDLS), malignant peripheral nerve sheath tumor (MPNST), myxofibrosarcoma (MFS), synovial sarcoma (SySa), undifferentiated pleomorphic sarcoma (UPS), angiosarcoma, pleomorphic liposarcoma (PLS), and others, including diverse range of rare STSs with definitive diagnosis other than described above.

### Statistical analysis

The primary endpoints were the occurrence of 1) local recurrence (LR), 2) distant metastasis (DM), and 3) disease specific death (DSD) at 2 years after surgery. LRFS or DMFS were defined as the period from the date of definitive surgery until the appearance of LR or DM, or until the last follow-up for patients without LR or DM, respectively. DSS was defined as the period from the date of definitive surgery until DSD, or until the last follow-up for survivors. Patients without these events or with lost to follow-up, or patients who died without LR or DM, or because of other causes, were censored at the last follow-up. The LRFS, DMFS and DSS were estimated using the Kaplan-Meier method, and comparisons were assessed using the log-rank test. Univariable and multivariable analyses were conducted using the Cox proportional hazards model. In the multivariable analysis, all the variables (age, sex, site, depth, size, histological diagnosis, histological grade, nodal metastasis, and surgical margin) were used as the independent variables with forced entry method. We determine the alpha level at 0.05 in this study.

### Nomogram

We developed the multivariate nomograms starting from Cox regression. The probabilities of each endpoint at 2 years after surgery were calculated for each patient with the Cox regression model underlying the nomogram. Then, these models were internally validated with 50 bootstrap samples to prevent over-fitting and obtain a relatively unbiased estimate. We evaluated the nomogram performance based on the concordance index and calibration plot. Concordance index indicates discrimination ability of nomogram to distinguish whether patients experience an interest event or not and calibration plot assesses how well the risk predicted by our nomogram fit the observed risk.

In addition, we performed decision curve analysis (DCA), a statistical method designed to evaluate the clinical usefulness of a prediction model without additional data [16]. DCA provides a relevant risk threshold for use of the model by comparing the net benefit of a model-assisted decision with those of treat-all and treat-none regardless of the model decision. When the risk threshold includes the probability that clinicians are motivated to perform a medical intervention, model is determined as a useful clinical model. The probability also coincide with a probability that harm of false positive intervention outweighs that of false negative non-intervention. For instance, if a patient has a LR probability of over 0.5, many clinicians would opt for some form of intervention, such as short-term follow-up using imaging or adjuvant therapy, whereas if the probability is 0.1, many clinicians would opt for simple observation. In this case, if the net benefit of the model-assisted decision exceeds those for treat-all and treat-none with a probability between 0.1 and 0.5, then the model is deemed clinically useful, because for many clinicians the risk threshold lies within this range.

Statistical analysis was performed using IBM SPSS version 19.0 (IBM SPSS, Armonk, NY, USA) and the nomograms and decision curves were built using R software version 3.0.1 (R Foundation for Statistical Computing, Vienna, Austria) with the rms and rmda library.

## Results

### Patients characteristics

We identified 2954 patients who fulfilled the inclusion criteria during 2006–2015. Among them, we excluded patients for whom any values with respect to age, sex, tumor size, grade, depth, location, histological diagnosis, presence or absence of nodal metastasis at diagnosis and surgical margin status were missing. As a result, our cohort consisted of 2827 patients, all of whom had a complete set of data for the variables described above. The clinicopathological characteristics of the cohort are summarized in Table 1. The median follow-up period was 16 months (range, 3–90 months). LR, DM and DSD occurred in 241 (8.5%), 554 (19.6%) and 230 (8.1%) patients over the study period, respectively (Additional file 1: Figure S1, Additional file 2: Figure S2 and Additional file 3: Figure S3).

**Table 1:** Clinicopathologic and treatment characteristics (*N* = 2827)

Characteristic		Number of patients (%)
Age, years	mean [SD]	60.2 [17.3]
	< 30	177 (6.3)
	30–49	572 (20.2)
	50–69	1094 (38.7)
	70-	984 (34.8)
Sex	Male	1573 (55.6)
	Female	1254 (44.4)
Site	Upper extremity	482 (17.0)
	Lower extremity	1871 (66.2)
	Trunk	474 (16.8)
Depth	Superficial	782 (27.7)
	Deep	2045 (72.3)
Histological diagnosis	MLS	349 (12.3)
	LMS	280 (9.9)
	DDLS	209 (7.4)
	MFS	130 (4.6)
	MPNST	347 (12.3)
	SySa	170 (6.0)
	UPS	779 (27.6)
	Angiosarcoma	21 (0.7)
	PLS	78 (2.8)
	Others	464 (16.4)
Histological grade	Low	546 (19.3)
	High	2281 (80.7)
Size, cm	mean [SD]	8.7 [5.7]
	5 cm<	749 (31.8)
	5 cm≤,< 10 cm	990 (42.1)
	10 cm≤	614 (26.1)
Nodal metastasis	Negative	2782 (98.4)
	Positive	45 (1.6)
Surgical margin	Negative	2674 (94.6)
	Positive	153 (5.4)
Chemotherapy	Neoadjuvant	226 (8.0)
	Adjuvant	603 (21.3)
	Not done	1998 (70.7)
Radiotherapy	Neoadjuvant	64 (2.3)
	Adjuvant	552 (19.5)
	Not done	2210 (78.2)
	NA	1 (0)

*CI* confidence interval, *MLS* myxoid liposarcoma, *LMS* leiomyosarcoma, *DDLS* dedifferentiated liposarcoma, *MPNST* malignant peripheral nerve sheath tumor, *MFS* myxofibrosarcoma, *SySa* synovial sarcoma, *UPS* undifferentiated pleomorphic sarcoma, *PLS* pleomorphic liposarcoma, *NA* not available

### Prognostic factors

Kaplan-Meier curves stratified by each variables and the results of log-rank test were exhibited in Additional file 1: Figure S1**,** Additional file 2: Figure S2 and Additional file 3: Figure S3. Nine prognostic factors selected based on clinical importance were significantly associated with each endpoints.

The results of the univariate and multivariate Cox proportional hazards models for LRFS are listed in Table 2. Multivariate analyses demonstrated that high-risk factors for LR were as follows: arising in the trunk (hazard ratio [HR], 2.55; 95% confidence interval [CI], 1.91–3.41; *P* < .001), large tumor size (≥ 5 cm and < 10 cm [HR, 1.95; 95% CI, 1.31–2.92; *P* = .001], ≥ 10 cm [HR, 3.50; 95% CI, 2.31–5.32; *P* < .001]), LMS [HR, 3.10; 95% CI, 1.43–6.69; *P* = .004], MFS [HR, 3.46; 95% CI, 1.51–7.94; *P* = .003], angiosarcoma [HR, 4.95; 95% CI, 1.32–18.58; *P* = .018], high grade (HR, 2.43; 95% CI, 1.48–3.99; *P* < .001) and positive margin (HR, 3.12; 95% CI, 2.22–4.37; *P* < .001).

**Table 2:** Cox proportional hazards models for LRFS

	Univariate analysis		Multivariate analysis	
	Hazard ratio (95% CI)	*P* value	Hazard ratio (95% CI)	*P* value
Age				
< 30	Reference		Reference	
30–49	0.80 (0.41–1.55)	0.505	0.82 (0.41–1.61)	0.557
50–69	1.26 (0.69–2.30)	0.450	0.99 (0.52–1.88)	0.971
70-	1.59 (0.88–2.90)	0.126	1.25 (0.65–2.39)	0.500
Sex				
Male	Reference		Reference	
Female	0.86 (0.66–1.11)	0.238	1.05 (0.81–1.36)	0.729
Site				
Lower extremity	Reference		Reference	
Upper extremity	1.31 (0.91–1.89)	0.142	1.46 (1.01–2.12)	0.045
Trunk	3.06 (2.32–4.05)	< 0.001	2.55 (1.91–3.41)	< 0.001
Depth				
Superficial	Reference		Reference	
Deep	2.16 (1.52–3.06)	< 0.001	1.33 (0.91–1.94)	0.139
Size				
5 cm<	Reference		Reference	
5 cm≤, < 10 cm	2.14 (1.45–3.16)	< 0.001	1.95 (1.31–2.92)	0.001
10 cm≤	4.19 (2.87–6.13)	< 0.001	3.50 (2.31–5.32)	< 0.001
Histological diagnosis				
MLS	Reference		Reference	
LMS	4.51 (2.15–9.47)	< 0.001	3.10 (1.43–6.69)	0.004
DDLS	7.20 (3.48–14.9)	< 0.001	2.58 (1.20–5.54)	0.015
MFS	5.77 (2.57–12.94)	< 0.001	3.46 (1.51–7.94)	0.003
MPNST	3.24 (1.53–6.84)	0.002	2.46 (1.13–5.34)	0.023
SySa	2.05 (0.81–5.16)	0.128	1.47 (0.57–3.79)	0.430
UPS	3.17 (1.57–6.37)	0.001	1.99 (0.96–4.13)	0.065
Angiosarcoma	7.34 (1.99–27.13)	0.003	4.95 (1.32–18.58)	0.018
PLS	1.99 (0.61–6.46)	0.252	1.14 (0.35–3.75)	0.828
Others	3.50 (1.70–7.22)	< 0.001	2.88 (1.37–6.07)	0.005
Histological grade				
Low	Reference		Reference	
High	2.93 (1.84–4.69)	< 0.001	2.43 (1.48–3.99)	< 0.001
Nodal meta				
Negative	Reference		Reference	
Positive	2.26 (1.12–4.57)	0.023	1.48 (0.70–3.10)	0.304
Surgical margin				
Negative	Reference		Reference	
Positive	4.29 (3.10–5.95)	< 0.001	3.12 (2.22–4.37)	< 0.001

*CI* confidence interval, *MLS* myxoid liposarcoma, *LMS* leiomyosarcoma, *DDLS* dedifferentiated liposarcoma, *MPNST* malignant peripheral nerve sheath tumor, *MFS* myxofibrosarcoma, *SySa* synovial sarcoma, *UPS* undifferentiated pleomorphic sarcoma, *PLS* pleomorphic liposarcoma

Table 3 shows the univariate and multivariate Cox proportional hazards models for DMFS. Multivariate analyses demonstrated that the high-risk factors for DM were as follows: large tumor size (≥ 5 cm and < 10 cm [HR, 2.02; 95% CI, 1.57–2.60; *P* < .001], ≥ 10 cm [HR, 3.39; 95% CI, 2.59–4.43; *P* < .001]), LMS [HR, 2.98; 95% CI, 2.00–4.45; *P* < .001], angiosarcoma [HR, 4.04; 95% CI, 1.78–9.15; *P* < .001], high grade (HR, 4.08; 95% CI, 2.74–6.09; *P* < .001) and positive nodal metastasis (HR, 2.06; 95% CI, 1.28–3.31; *P* = .003).

**Table 3:** Cox proportional hazards models for DMFS

	Univariate analysis		Multivariate analysis	
	Hazard ratio (95% CI)	*P* value	Hazard ratio (95% CI)	*P* value
Age				
< 30	Reference		Reference	
30–49	0.68 (0.45–1.03)	0.069	0.67 (0.43–1.02)	0.062
50–69	1.16 (0.80–1.68)	0.435	0.94 (0.63–1.41)	0.777
70-	1.27 (0.88–1.84)	0.202	1.02 (0.68–1.54)	0.909
Sex				
Male	Reference		Reference	
Female	0.79 (0.67–0.94)	0.007	0.89 (0.74–1.05)	0.166
Site				
Lower extremity	Reference		Reference	
Upper extremity	0.97 (0.77–1.22)	0.773	1.07 (0.85–1.35)	0.576
Trunk	1.12 (0.90–1.39)	0.314	1.05 (0.83–1.31)	0.696
Depth				
Superficial	Reference		Reference	
Deep	1.92 (1.54–2.39)	< 0.001	1.34 (1.06–1.70)	0.015
Size				
5 cm<	Reference		Reference	
5 cm≤, < 10 cm	2.13 (1.67–2.72)	< 0.001	2.02 (1.57–2.60)	< 0.001
10 cm≤	3.46 (2.71–4.42)	< 0.001	3.39 (2.59–4.43)	< 0.001
Histological diagnosis				
MLS	Reference		Reference	
LMS	3.79 (2.59–5.54)	< 0.001	2.98 (2.00–4.45)	< 0.001
DDLS	1.40 (0.87–2.26)	0.166	0.70 (0.42–1.15)	0.160
MFS	2.31 (1.42–3.76)	< 0.001	1.87 (1.14–3.08)	0.014
MPNST	1.29 (0.84–1.99)	0.245	1.19 (0.76–1.86)	0.454
SySa	1.52 (0.92–2.49)	0.099	1.35 (0.81–2.27)	0.250
UPS	2.33 (1.63–3.32)	< 0.001	1.64 (1.12–2.38)	0.011
Angiosarcoma	4.04 (1.80–9.07)	< 0.001	4.04 (1.78–9.15)	< 0.001
PLS	2.27 (1.29–3.99)	0.004	1.38 (0.78–2.45)	0.274
Others	1.81 (1.23–2.67)	0.003	1.91 (1.28–2.86)	0.002
Histological grade				
Low	Reference		Reference	
High	5.19 (3.52–7.64)	< 0.001	4.08 (2.74–6.09)	< 0.001
Nodal metastasis				
Negative	Reference		Reference	
Positive	2.69 (1.70–4.25)	< 0.001	2.06 (1.28–3.31)	0.003
Surgical margin				
Negative	Reference		Reference	
Positive	1.35 (0.98–1.88)	0.067	1.15 (0.83–1.6)	0.404

*CI* confidence interval, *MLS* myxoid liposarcoma, *LMS* leiomyosarcoma, *DDLS* dedifferentiated liposarcoma, *MPNST* malignant peripheral nerve sheath tumor, *MFS* myxofibrosarcoma, *SySa* synovial sarcoma, *UPS* undifferentiated pleomorphic sarcoma, *PLS* pleomorphic liposarcoma

Table 4 shows the univariate and multivariate Cox proportional hazards models for DSS. Multivariate analyses demonstrated that the high-risk factors of DSD were as follows: large tumor size (≥ 5 cm and < 10 cm [HR, 2.19; 95% CI, 1.41–3.39; *P* < .001], ≥ 10 cm [HR, 4.73; 95% CI, 3.03–7.38; *P* < .001]), LMS [HR, 2.94; 95% CI, 1.41–6.10; *P* = .004], MFS [HR, 3.21; 95% CI, 1.43–7.20; *P* = .005], angiosarcoma [HR, 11.40; 95% CI, 4.05–32.10; *P* < .001], high grade (HR, 5.37; 95% CI, 2.61–11.04; *P* < .001), and positive margin (HR, 1.54; 95% CI, 1.00–2.36; *P* = .048).

**Table 4:** Cox proportional hazards models for DSS

	Univariate analysis		Multivariate analysis	
	Hazard ratio (95% CI)	*P* value	Hazard ratio (95% CI)	*P* value
Age				
< 30	Reference		Reference	
30–49	0.72 (0.34–1.51)	0.383	0.69 (0.32–1.46)	0.332
50–69	1.23 (0.64–2.37)	0.541	0.93 (0.46–1.87)	0.844
70-	2.21 (1.16–4.21)	0.016	1.72 (0.86–3.43)	0.127
Sex				
Male	Reference		Reference	
Female	0.79 (0.60–1.03)	0.076	0.90 (0.68–1.17)	0.426
Site				
Lower extremity	Reference		Reference	
Upper extremity	1.10 (0.77–1.56)	0.616	1.21 (0.84–1.74)	0.300
Trunk	1.54 (1.12–2.11)	0.007	1.41 (1.01–1.95)	0.041
Depth				
Superficial	Reference		Reference	
Deep	2.48 (1.70–3.62)	< 0.001	1.58 (1.06–2.38)	0.026
Size				
5 cm<	Reference		Reference	
5 cm≤, < 10 cm	2.41 (1.57–3.68)	< 0.001	2.19 (1.41–3.39)	< 0.001
10 cm≤	5.20 (3.44–7.85)	< 0.001	4.73 (3.03–7.38)	< 0.001
Histological diagnosis				
MLS	Reference		Reference	
LMS	4.66 (2.31–9.39)	< 0.001	2.94 (1.41–6.10)	0.004
DDLS	2.66 (1.2–5.85)	0.015	0.92 (0.41–2.10)	0.851
MFS	4.62 (2.1–10.18)	< 0.001	3.21 (1.43–7.20)	0.005
MPNST	1.91 (0.89–4.1)	0.098	1.37 (0.62–3.03)	0.433
SySa	1.03 (0.35–3.01)	0.959	0.88 (0.29–2.62)	0.811
UPS	3.48 (1.8–6.73)	< 0.001	1.90 (0.95–3.78)	0.070
Angiosarcoma	13.51 (4.91–37.19)	< 0.001	11.40 (4.05–32.10)	< 0.001
PLS	1.80 (0.56–5.74)	0.320	0.87 (0.27–2.80)	0.812
Others	3.34 (1.68–6.65)	< 0.001	2.89 (1.42–5.89)	0.003
Histological grade				
Low	Reference		Reference	
High	6.88 (3.40–13.92)	< 0.001	5.37 (2.61–11.04)	< 0.001
Nodal metastasis				
Negative	Reference		Reference	
Positive	2.95 (1.56–5.55)	< 0.001	1.95 (1.00–3.80)	0.051
Surgical margin				
Negative	Reference		Reference	
Positive	2.15 (1.42–3.25)	< 0.001	1.54 (1.01–2.36)	0.048

*CI* confidence interval, *MLS* myxoid liposarcoma, *LMS* leiomyosarcoma, *DDLS* dedifferentiated liposarcoma, *MPNST* malignant peripheral nerve sheath tumor, *MFS* myxofibrosarcoma, *SySa* synovial sarcoma, *UPS* undifferentiated pleomorphic sarcoma, *PLS* pleomorphic liposarcoma

### Availability of developed nomograms

**Fig. 1:**
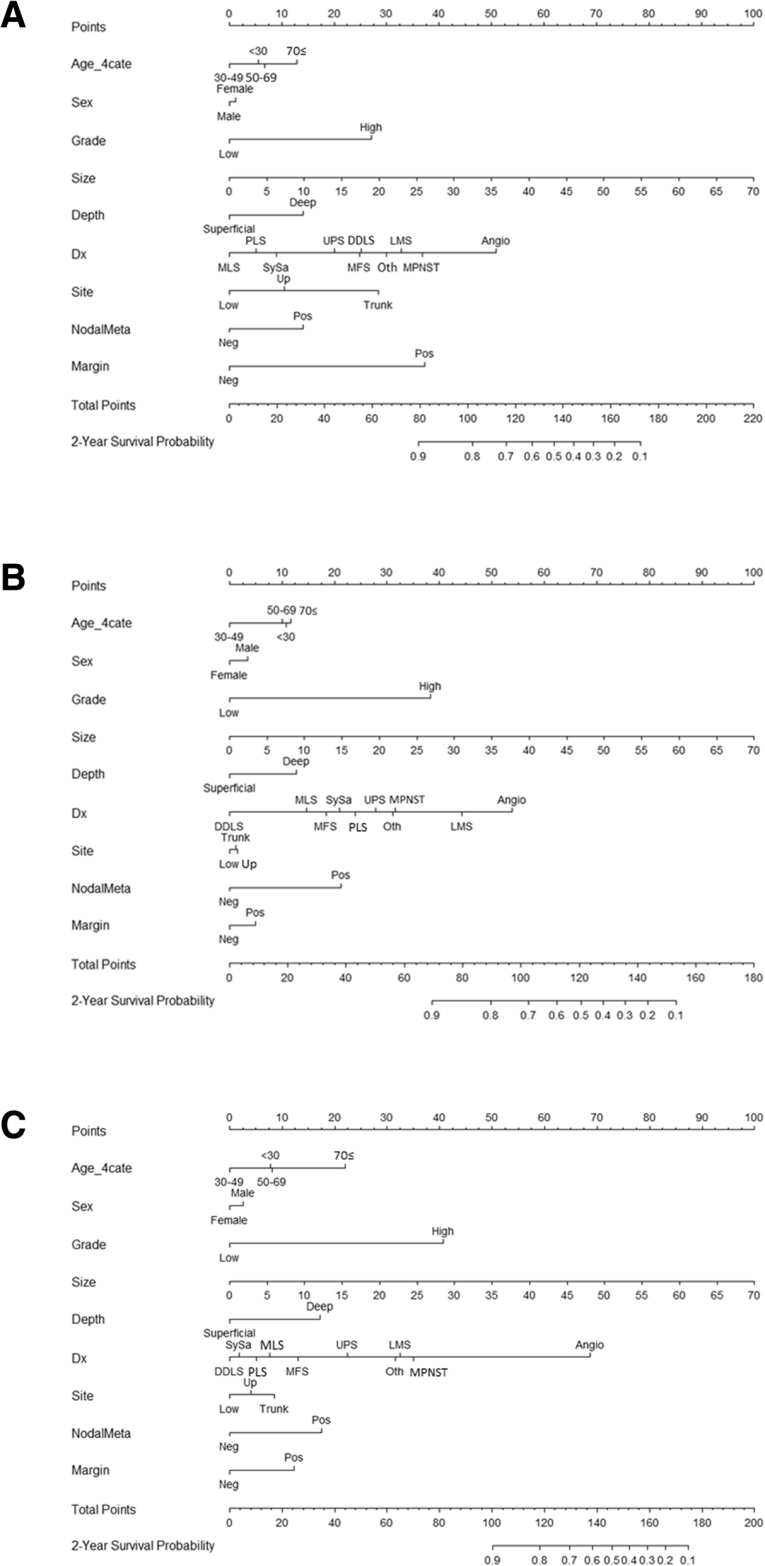
Nomograms predicting the probability of LRFS (**a**), DMFS (**b**), and DSS (**c**) at 2 years after surgery. For use, the value for each variable in an individual patient is selected on the scale, and a line is drawn straight up from there to the Points axis to establish the corresponding score. All the scores are summed, and the total score is plotted on the Total Points line. A line is then drawn straight down to the 2-Year Survival Probability axis to obtain the probability. Dx, histological diagnosis; MLS, myxoid liposarcoma; LMS, leiomyosarcoma; DDLS, dedifferentiated liposarcoma; MPNST, malignant peripheral nerve sheath tumor; MFS, myxofibrosarcoma; SySa, synovial sarcoma; UPS, undifferentiated pleomorphic sarcoma; Angio, angiosarcoma,; PLS, pleomorphic liposarcoma; Oth, others; Neg, negative; Pos, positive

**Fig. 2:**
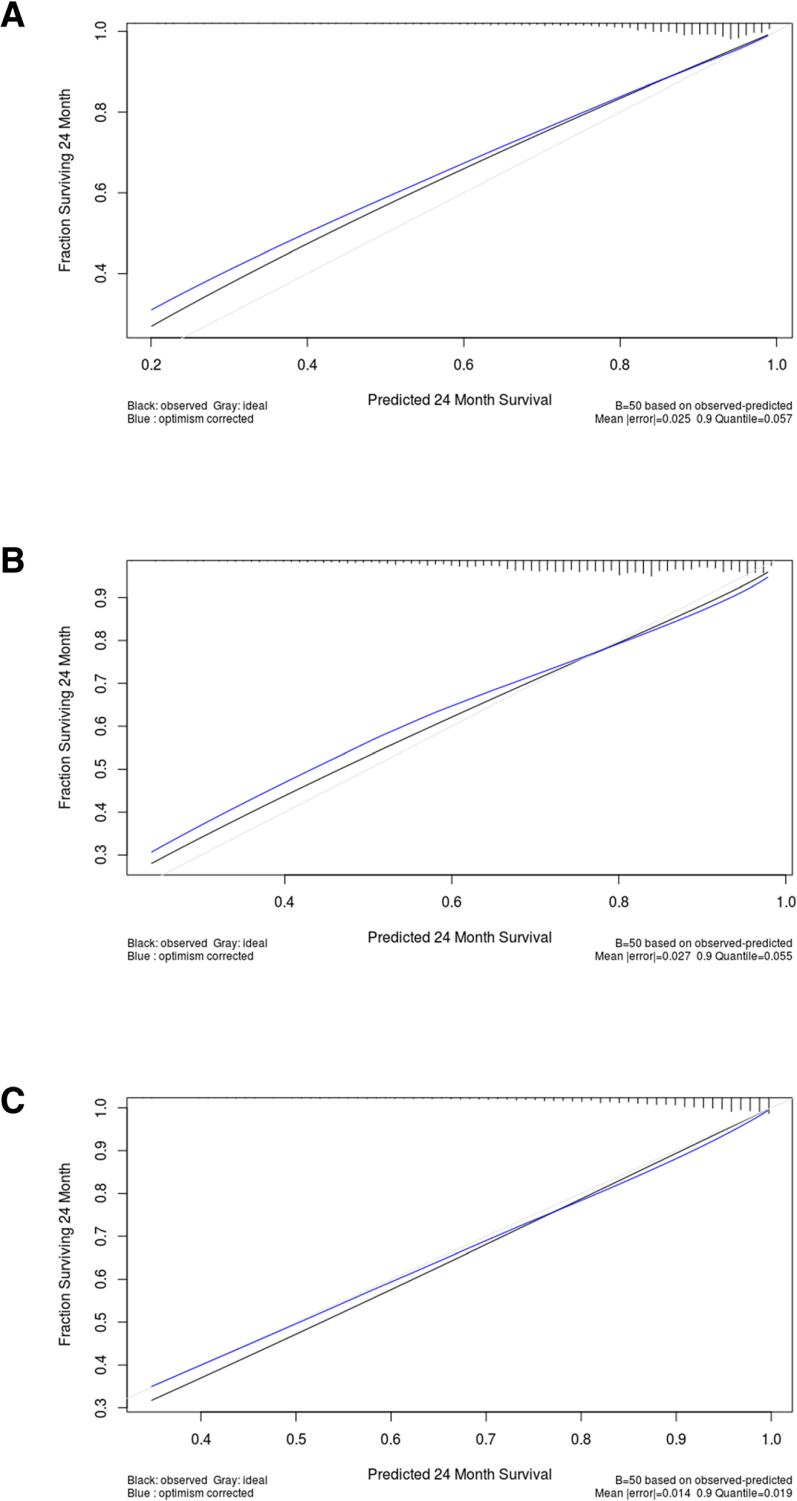
Calibration plots for internal validation of the LRFS (**a**), DMFS (**b**), and DSS (**c**) nomograms. Gray, ideal; black, observed; blue, bias corrected

We developed nomograms to predict LRFS, DMFS, and DSS at 2 years after surgery based on the weighted coefficients for each of the values obtained by Cox regression (Fig. 1a, b and c). The calibration plots for internal validation of these nomograms are shown in Fig. 2a, b and c. Concordance indices for the LRFS, DMFS and DSS nomograms were 0.73, 0.70 and 0.75, respectively, suggesting high predictive accuracy. Furthermore, in order to assess the clinical utility of this nomogram, we conducted DCA. The results of DCA demonstrated that use of these nomograms for clinical decision-making was more efficient than assuming that all patients would be treated or not treated, with a risk threshold ranging from 0.05 to 0.8, from 0.05 to 0.4, and from 0.05 to 0.3, respectively (Additional file 4: Figure S4).

## Discussion

Recently, nomograms have been accepted for prognostication of sarcoma and other major cancers [9, 11, 17] because they enable clinicians to predict accurately any individual patient’s outcome, as well as being very user-friendly. However, nomograms need to be updated over time in accordance with the development of new treatments or changes in diagnostic criteria that may affect the course of the disease. Nevertheless, there are currently few nomograms for STSs based on the recent WHO histological classification [8, 10, 12]. Moreover, to our knowledge, no nomograms for STSs have been based on Asian cohorts. In the present study, we developed nomograms for prediction of LRFS, DMFS, and DSS in patients with primary STSs diagnosed on the basis of the recent WHO classification after definitive surgery employing a large cohort of Japanese patients in the BSTT registry. In addition, we confirmed the predictive accuracy and clinical utility of these nomograms by internal validation and DCA analysis, respectively.

We found that the LR, DM, and DSD incidence at 2 years after surgery were 8.5, 19.6 and 8.1%, respectively, being consistent with previous studies [3, 4]. In addition, histological diagnosis, grade, and tumor size have generally been accepted as predictive factors for STS in previous large-scale retrospective studies [2–13], and as expected, these factors were independent prognostic factors for all of the outcomes in our multivariate analyses (Tables 2-4). Furthermore, in agreement with previous studies, the surgical margin also strongly influenced the LRFS in our dataset [18, 19]. Because not only the variables using TNM staging but also other variables influenced each outcomes, our prediction models can stratify the patients with the same TNM staging into the risks of each outcome. For example, we compared two cases in stage III (seventh edition of AJCC staging system) as follows: 1) a 46-year-old man who presented with a 6 cm and deep-seated mass in his lower extremity. The tumor is resected with negative margin and diagnosed as high grade DDLS and; 2) a 78-year-old woman presented with 20 cm and deep-seated mass in her lower extremity. The tumor is also resected with negative margin and diagnosed as high grade LMS. The 2-year probabilities of DMFS in our nomogram were > 90 and 43%, respectively. Actually, the former case did not experience a distant metastasis, while the latter case experienced it at 11 weeks after definitive surgery.

One of the unique advantages of our nomograms is that they incorporate nodal metastasis status as a prognostic factor in the predictive models. No predictive nomograms reported to date have incorporated nodal metastasis despite its significant impact on survival, being one of the factors in the AJCC staging system, probably due to the rare incidence of nodal metastasis without distant metastasis, comprising only 2.1% of all STS cases [20, 21]. The use of a national database in our study enabled us to collect a large number of STS cases as a training data set for developing predictive nomograms, enabling us to incorporate rare but important prognostic factors such as nodal metastasis and thus develop a more accurate predictive model. Another advantage of our nomograms is that they can predict three endpoints, unlike previously published nomograms [8–12], in terms of major progression events occurring during the clinical course of cancer, thus providing physicians with more detailed information about the future clinical course, and allowing better clinical care to be offered.

We acknowledge that the present study had some limitations. First, although adjuvant radiotherapy (RT) in addition to surgical resection is accepted as the standard treatment for STS in Western countries based on the favorable results of randomized trials [22, 23], we were unable to find any significant advantage of adjuvant RT for patients with STS. One of the main reasons for this conflicting result may have been the difference in the indications for adjuvant RT, which is used in Japan only for a small proportion of STS patients who have a higher risk of local relapse. In fact only 22% of patients in our dataset underwent adjuvant radiotherapy, compared with 32–82% in previous reports from Western countries [3, 4, 6, 9, 11]. Therefore, we investigated the differences in background characteristics between patients undergoing and not undergoing adjuvant RT (Additional file 5: Table S1). As expected, patients undergoing adjuvant RT showed significant differences in histological grade, tumor size, surgical margin, and nodal metastasis, suggesting that patients with a higher risk of local relapse underwent adjuvant RT in Japan. Therefore, there will be a need to externally validate our nomograms using specific patient populations before they can be recommended for clinical use even in Western countries, where therapeutic strategies are different. Second, as the BSTT data are collected from all hospitals across Japan, the differences in the perioperative management and the follow-up interval to check tumor recurrence after definitive surgery may vary among hospitals. In addition, some cases with pathological misdiagnosis may be included in the BSTT Registry because the rate of discordance of pathological diagnosis in STS is high [24]. These issues may result in information bias. Third, the results of DCA analysis for the LRFS model were widely acceptable in the risk probability range, whereas those for the DMFS and DSS model were available only from the low to middle risk probability range (Additional file 4: Figure S4). However, in terms of decision-making, a low or middle risk probability of DM or DSD is a more important issue than high risk, because there is no risk threshold at high probability. Therefore, our nomograms achieved a sufficient level of clinical usefulness. Fourth, because the majority of cases in the BSTT Registry were registered from specialized cancer hospitals, the frequency of severe cases in the BSTT Registry may be higher than that of population-based data. In addition, the BSTT Registry data is operated by JOA which means most of the cases are patients treated by musculoskeletal surgeons. Therefore, cases treated in other specialities would not be included in the registry: ex., advanced cases treated only by medical oncologist or sarcoma arising from the retroperitoneum or peritoneal cavity which can be treated by abdominal surgeon. For these reasons, there may be the difference of patient backgrounds between the BSTT Registry and the population-based database. Finally, our nomograms were able to predict only the 2-year probability of LRFS, DMFS, and DSS, because the follow-up period for BSTT data is still relatively short. Data for longer follow-up periods will therefore be required in order to develop a predictive model for estimation of survival at 5 or 10 years.

## Conclusion

In conclusion, we have created nomograms for prediction of LRFS, DMFS, and DSS at 2 years after definitive surgery for patients with localized STSs in the trunk and extremity. We have confirmed the predictive accuracy and clinical utility of these nomograms by internal validation and DCA analysis, respectively. These nomograms can help clinicians to make decisions regarding adjuvant therapy or the interval of follow-up imaging, and also as a guide for patient counselling.

## Additional files

Additional file 1: **Figure S1.** Kaplan-Meier curves stratified by predictive variables on LRFS: (A) overall, (B) age (< 30, 30–49, 50–69, > 70), (C) sex,, (D) tumor site (lower extremity, upper extremity, trunk), (E) tumor depth (superficial, deep), (F) tumor size (< 5 cm, ≥5 cm and < 10 cm, ≥10 cm), (G) Histological diagnosis (MLS, LMS, DDLS, MPNST, MFS, SySa, UPS, Angio, PLS, Others), (H) histological grade (low, high), (I) nodal metastasis (negative, positive), and (J) surgical margin (negative, positive). (TIF 5546 kb)

Additional file 2: **Figure S2.** Kaplan-Meier curves stratified by predictive variables on DMFS: (A) overall, (B) age (< 30, 30–49, 50–69, > 70), (C) sex,, (D) tumor site (lower extremity, upper extremity, trunk), (E) tumor depth (superficial, deep), (F) tumor size (< 5 cm, ≥5 cm and < 10 cm, ≥10 cm), (G) Histological diagnosis (MLS, LMS, DDLS, MPNST, MFS, SySa, UPS, Angio, PLS, Others), (H) histological grade (low, high), (I) nodal metastasis (negative, positive), and (J) surgical margin (negative, positive). (TIF 5645 kb)

Additional file 3: **Figure S3.** Kaplan-Meier curves stratified by predictive variables on DSS: (A) overall, (B) age (< 30, 30–49, 50–69, > 70), (C) sex,, (D) tumor site (lower extremity, upper extremity, trunk), (E) tumor depth (superficial, deep), (F) tumor size (< 5 cm, ≥5 cm and < 10 cm, ≥10 cm), (G) Histological diagnosis (MLS, LMS, DDLS, MPNST, MFS, SySa, UPS, Angio, PLS, Others), (H) histological grade (low, high), (I) nodal metastasis (negative, positive), and (J) surgical margin (negative, positive). (TIF 5373 kb)

Additional file 4: **Figure S4.** Decision curves for LRFS (A), DMFS (B), and DSS (C) at 2 years after surgery. Solid bold line, an assumed strategy of treating no patients; solid thin line, an assumed strategy of treating all patients; red line, a strategy of treating patients according to the nomogram predictions. (TIF 5820 kb)

Additional file 5: **Table S1.** Logistic regression analysis for factors associated with receiving RT. (DOCX 18 kb)

## Data Availability

The datasets used and/or analyzed during the current study are available from the corresponding author on reasonable request.

## Abbreviations

AJCC: American Joint Committee on Cancer
BSTT: Bone and Soft Tissue Tumor
CI: Confidence interval;
DCA: Decision curve analysis
DDLS: Dedifferentiated liposarcoma
DM: Distant metastasis
DMFS: Distant metastasis-free survival
DSD: Disease specific death
DSS: Disease-specific survival
HR: Hazard ratio
JOA: Japanese orthopaedic association
LMS: Leiomyosarcoma
LR: Local recurrence
LRFS: Local recurrence-free survival
MFS: Myxofibrosarcoma
MLS: Myxoid liposarcoma
MPNST: Malignant peripheral nerve sheath tumor
PLS: Pleomorphic liposarcoma
RT: Radiotherapy
STS: Soft tissue sarcoma
SySa: Synovial sarcoma
UPS: Undifferentiated pleomorphic sarcoma
WHO: World Health Organization

## References

[1] Amin MB, Edge SB, Greene FL, Byrd DR, Brookland RK, Washington MK (2017). American joint committee on Cancer. AJCC Cancer staging manual.

[2] Ogura K, Higashi T, Kawai A (2017). Statistics of soft-tissue sarcoma in Japan: report from the bone and soft tissue tumor registry in Japan. J Orthop Sci.

[3] Gronchi A, Lo Vullo S, Colombo C, Collini P, Stacchiotti S, Mariani L (2010). Extremity soft tissue sarcoma in a series of patients treated at a single institution: local control directly impacts survival. Ann Surg.

[4] Italiano A, Le Cesne A, Mendiboure J, Blay JY, Piperno-Neumann S, Chevreau C (2014). Prognostic factors and impact of adjuvant treatments on local and metastatic relapse of soft-tissue sarcoma patients in the competing risks setting. Cancer.

[5] Zagars GK, Ballo MT, Pisters PW, Pollock RE, Patel SR, Benjamin RS (2003). Prognostic factors for patients with localized soft-tissue sarcoma treated with conservation surgery and radiation therapy: an analysis of 1225 patients. Cancer.

[6] Maretty-Nielsen K, Aggerholm-Pedersen N, Safwat A, Jørgensen PH, Hansen BH, Baerentzen S (2014). Prognostic factors for local recurrence and mortality in adult soft tissue sarcoma of the extremities and trunk wall: a cohort study of 922 consecutive patients. Acta Orthop.

[7] van Praag VM, Rueten-Budde AJ, Jeys LM, Laitinen MK, Pollock R, Aston W (2017). A prediction model for treatment decisions in high-grade extremity soft-tissue sarcomas: personalised sarcoma care (PERSARC). Eur J Cancer.

[8] Kattan MW, Leung DH, Brennan MF (2002). Postoperative nomogram for 12-year sarcoma-specific death. J Clin Oncol.

[9] Gronchi A, Miceli R, Shurell E, Eilber FC, Eilber FR, Anaya DA (2013). Outcome prediction in primary resected retroperitoneal soft tissue sarcoma: histology-specific overall survival and disease-free survival nomograms built on major sarcoma center data sets. J Clin Oncol.

[10] Cahlon O, Brennan MF, Jia X, Qin LX, Singer S, Alektiar KM (2012). A postoperative nomogram for local recurrence risk in extremity soft tissue sarcomas after limb-sparing surgery without adjuvant radiation. Ann Surg.

[11] Callegaro D, Miceli R, Bonvalot S, Ferguson P, Strauss DC, Levy A (2016). Development and external validation of two nomograms to predict overall survival and occurrence of distant metastases in adults after surgical resection of localised soft-tissue sarcomas of the extremities: a retrospective analysis. Lancet Oncol.

[12] Mariani L, Miceli R, Kattan MW, Brennan MF, Colecchia M, Fiore M (2005). Validation and adaptation of a nomogram for predicting the survival of patients with extremity soft tissue sarcoma using a three-grade system. Cancer.

[13] Kattan MW, Hess KR, Amin MB, Lu Y, Moons KG, Gershenwald JE (2016). American joint committee on Cancer acceptance criteria for inclusion of risk models for individualized prognosis in the practice of precision medicine. CA Cancer J Clin.

[14] Fletcher CDM, Bridge JA, Hogendoorn CW, Mertens F (2013). WHO classification of tumours of soft tissue and bone.

[15] Ogura K, Higashi T, Kawai A (2017). Statistics of bone sarcoma in Japan: report from the bone and soft tissue tumor registry in Japan. J Orthop Sci.

[16] Vickers AJ, Elkin EB (2006). Decision curve analysis: a novel method for evaluating prediction models. Med Decis Mak.

[17] Ogura K, Fujiwara T, Yasunaga H, Matsui H, Jeon DG, Cho WH (2015). Development and external validation of nomograms predicting distant metastases and overall survival after neoadjuvant chemotherapy and surgery for patients with nonmetastatic osteosarcoma: a multi-institutional study. Cancer.

[18] Willeumier J, Fiocco M, Nout R, Dijkstra S, Aston W, Pollock R (2015). High-grade soft tissue sarcomas of the extremities: surgical margins influence only local recurrence not overall survival. Int Orthop.

[19] Stojadinovic A, Leung DH, Hoos A, Jaques DP, Lewis JJ, Brennan MF (2002). Analysis of the prognostic significance of microscopic margins in 2,084 localized primary adult soft tissue sarcomas. Ann Surg.

[20] Johannesmeyer D, Smith V, Cole DJ, Esnaola NF, Camp ER (2013). The impact of lymph node disease in extremity soft-tissue sarcomas: a population-based analysis. Am J Surg.

[21] Keung EZ, Chiang YJ, Voss RK, Cormier JN, Torres KE, Hunt KK (2018). Defining the incidence and clinical significance of lymph node metastasis in soft tissue sarcoma. Eur J Surg Oncol.

[22] Yang JC, Chang AE, Baker AR, Sindelar WF, Danforth DN, Topalian SL (1998). Randomized prospective study of the benefit of adjuvant radiation therapy in the treatment of soft tissue sarcomas of the extremity. J Clin Oncol.

[23] Pisters PW, Leung DH, Woodruff J, Shi W, Brennan MF (1996). Analysis of prognostic factors in 1,041 patients with localized soft tissue sarcomas of the extremities. J Clin Oncol.

[24] Ray-Coquard I, Montesco MC, Coindre JM, Dei Tos AP, Lurkin A, Ranchère-Vince D (2012). Sarcoma: concordance between initial diagnosis and centralized expert review in a population-based study within three European regions. Ann Oncol.

