# Supplementary figures and images for: Development of nomograms for prognostication of patients with primary soft tissue sarcomas of the trunk and extremity: report from the Bone and Soft Tissue Tumor Registry in Japan
Source: BMC Cancer. 2019 Jul 4;19:657. doi: 10.1186/s12885-019-5875-y (PMC6610844; doi:10.1186/s12885-019-5875-y)

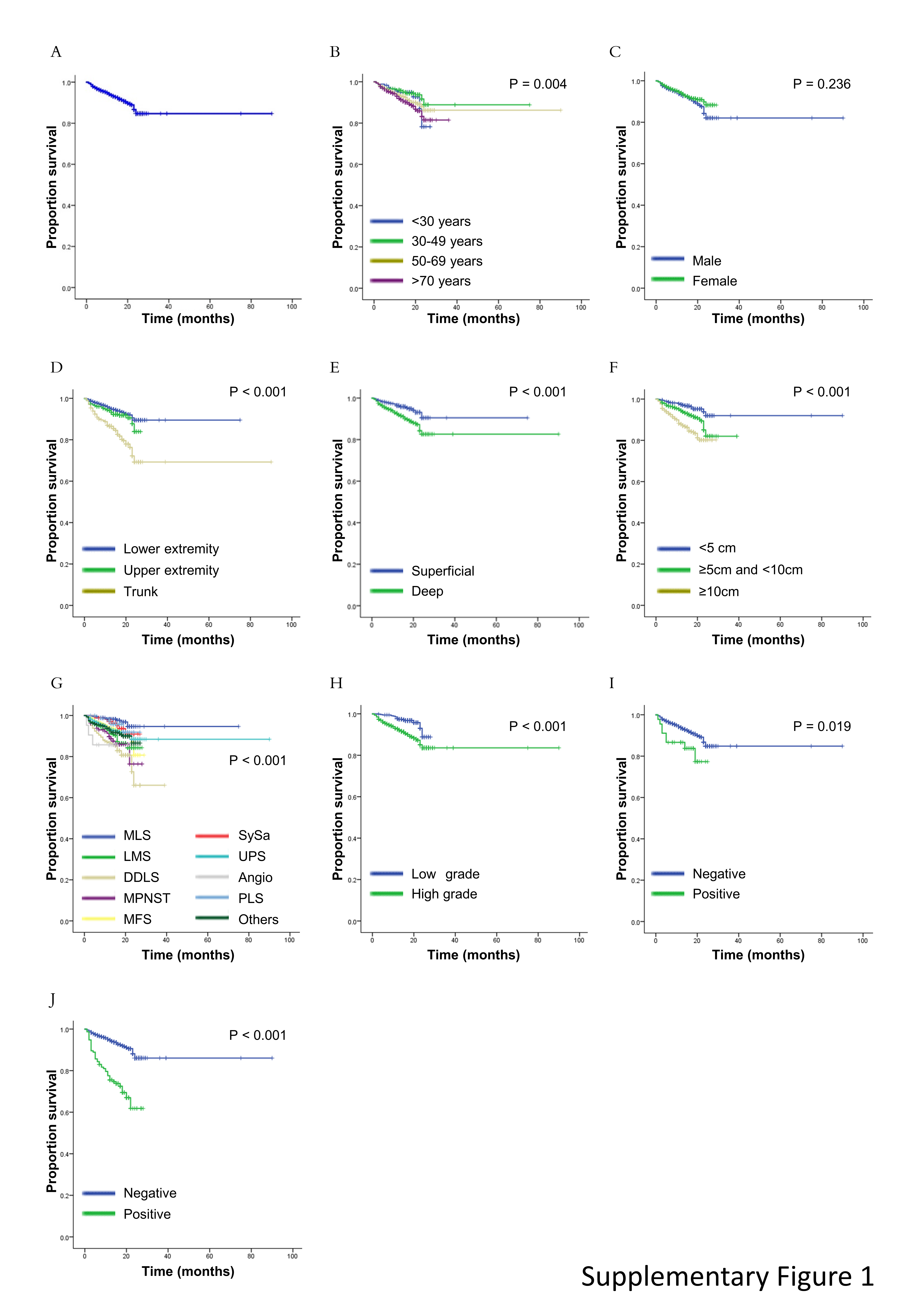

Supplement: Supplementary file 1 — Figure S1. Kaplan-Meier curves stratified by predictive variables on LRFS: (A) overall, (B) age (< 30, 30–49, 50–69, > 70), (C) sex,, (D) tumor site (lower extremity, upper extremity, trunk), (E) tumor depth (superficial, deep), (F) tumor size (< 5 cm, ≥5 cm and < 10 cm, ≥10 cm), (G) Histological diagnosis (MLS, LMS, DDLS, MPNST, MFS, SySa, UPS, Angio, PLS, Others), (H) histological grade (low, high), (I) nodal metastasis (negative, positive), and (J) surgical margin (negative, positive). (TIF 5546 kb) [file 12885_2019_5875_MOESM1_ESM.tif]

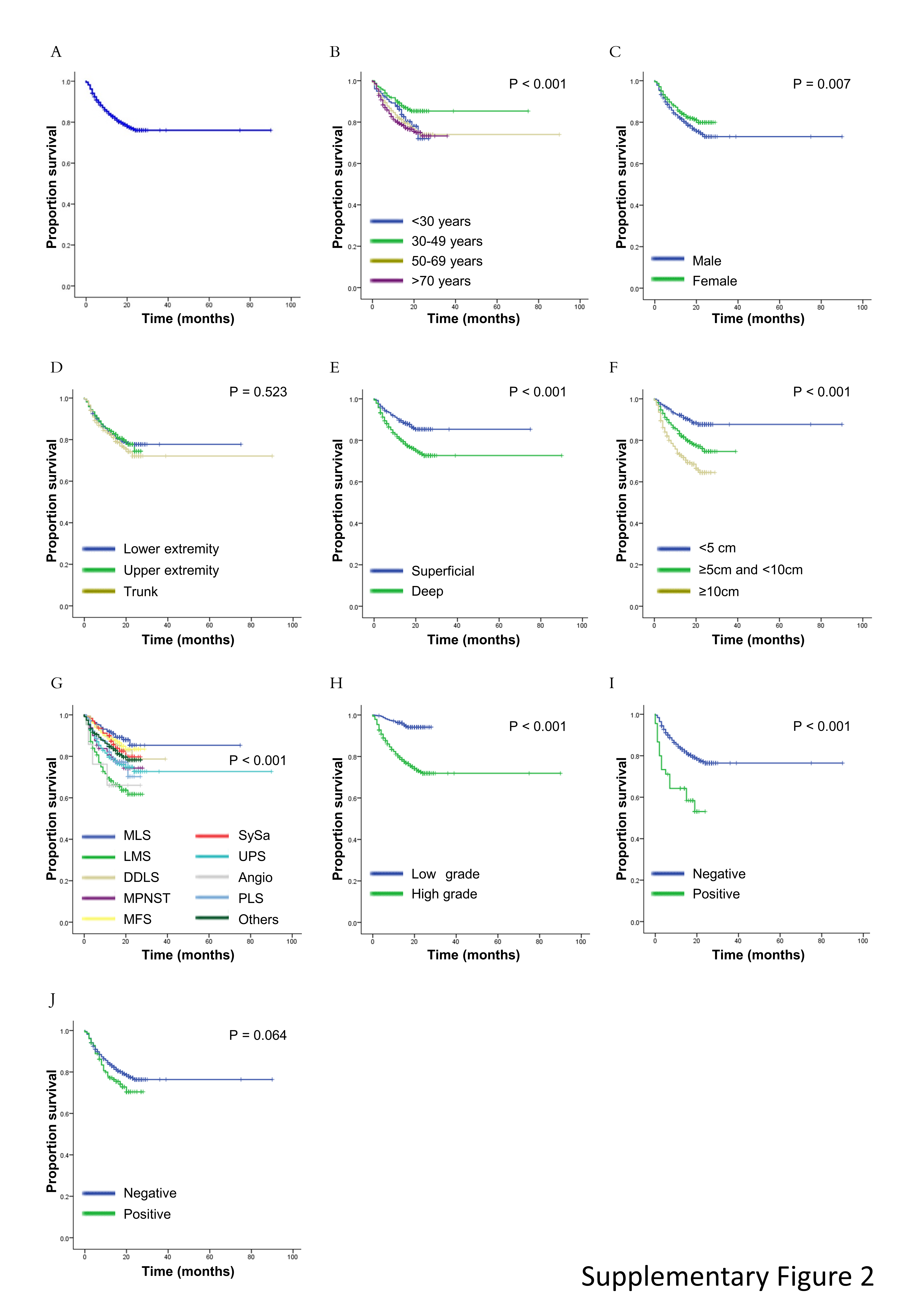

Supplement: Supplementary file 2 — Figure S2. Kaplan-Meier curves stratified by predictive variables on DMFS: (A) overall, (B) age (< 30, 30–49, 50–69, > 70), (C) sex,, (D) tumor site (lower extremity, upper extremity, trunk), (E) tumor depth (superficial, deep), (F) tumor size (< 5 cm, ≥5 cm and < 10 cm, ≥10 cm), (G) Histological diagnosis (MLS, LMS, DDLS, MPNST, MFS, SySa, UPS, Angio, PLS, Others), (H) histological grade (low, high), (I) nodal metastasis (negative, positive), and (J) surgical margin (negative, positive). (TIF 5645 kb) [file 12885_2019_5875_MOESM2_ESM.tif]

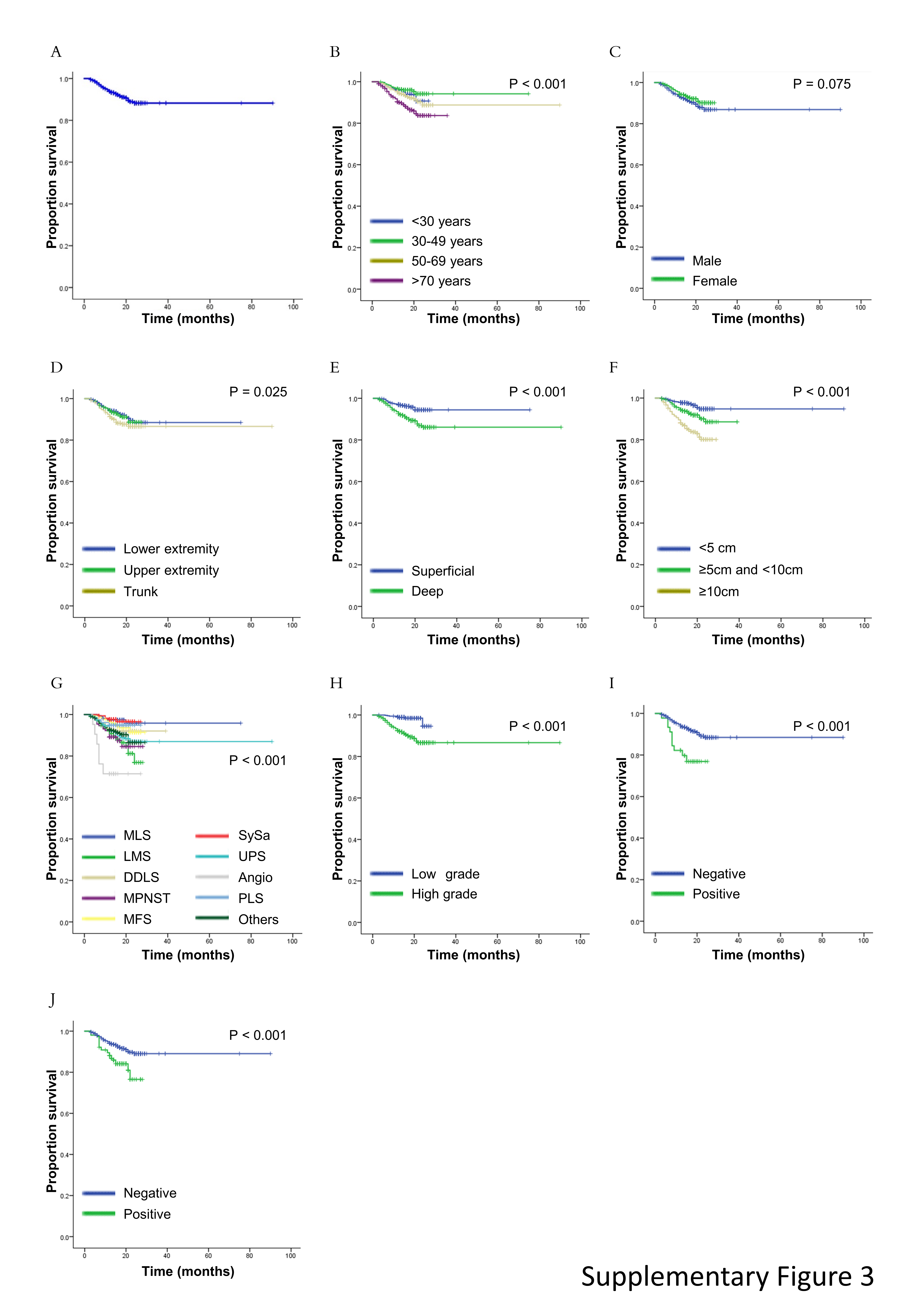

Supplement: Supplementary file 3 — Figure S3. Kaplan-Meier curves stratified by predictive variables on DSS: (A) overall, (B) age (< 30, 30–49, 50–69, > 70), (C) sex,, (D) tumor site (lower extremity, upper extremity, trunk), (E) tumor depth (superficial, deep), (F) tumor size (< 5 cm, ≥5 cm and < 10 cm, ≥10 cm), (G) Histological diagnosis (MLS, LMS, DDLS, MPNST, MFS, SySa, UPS, Angio, PLS, Others), (H) histological grade (low, high), (I) nodal metastasis (negative, positive), and (J) surgical margin (negative, positive). (TIF 5373 kb) [file 12885_2019_5875_MOESM3_ESM.tif]

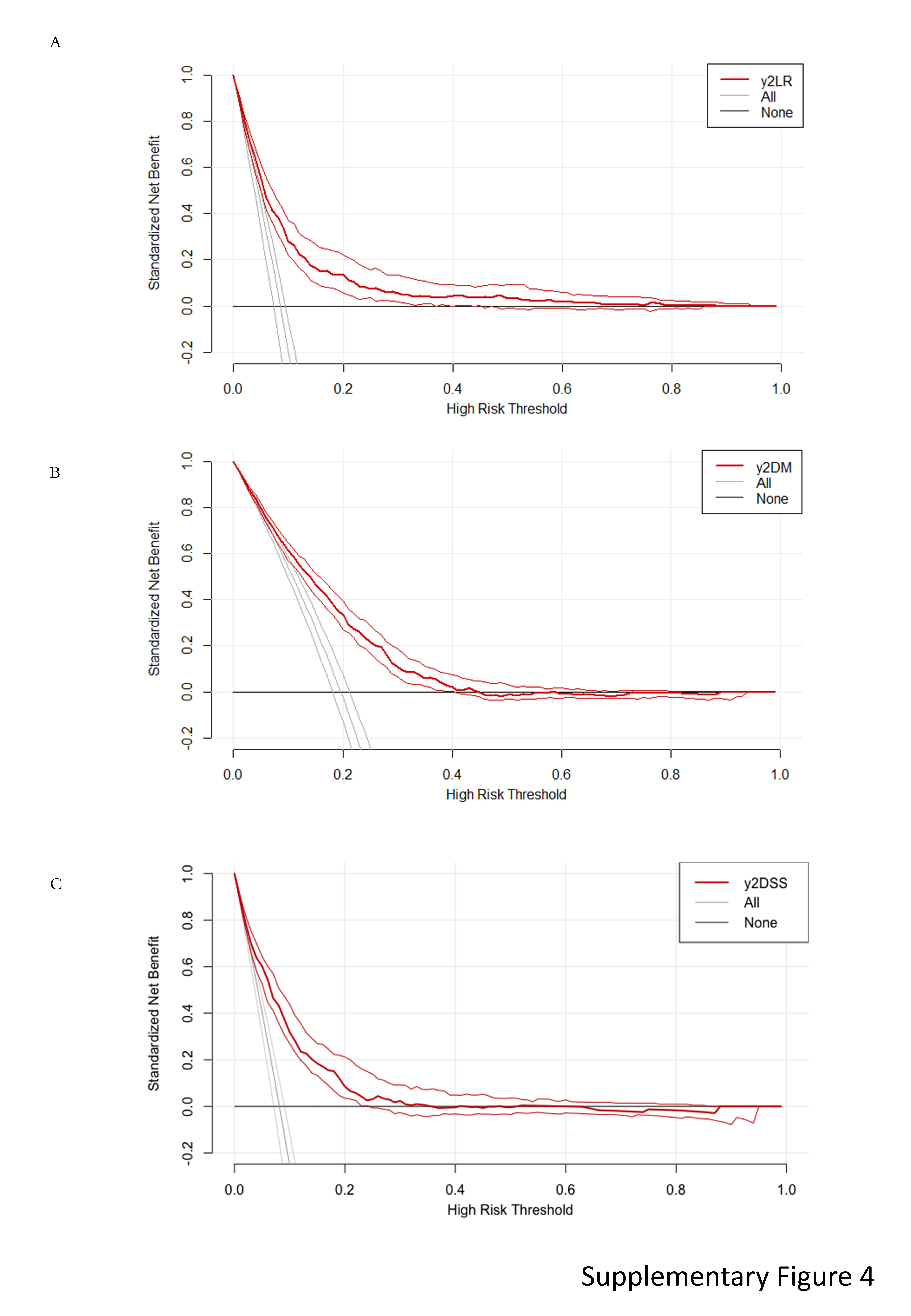

Supplement: Supplementary file 4 — Figure S4. Decision curves for LRFS (A), DMFS (B), and DSS (C) at 2 years after surgery. Solid bold line, an assumed strategy of treating no patients; solid thin line, an assumed strategy of treating all patients; red line, a strategy of treating patients according to the nomogram predictions. (TIF 5820 kb) [file 12885_2019_5875_MOESM4_ESM.tif]
