# Supplementary material for: Development of nomograms for prognostication of patients with primary soft tissue sarcomas of the trunk and extremity: report from the Bone and Soft Tissue Tumor Registry in Japan
Source: BMC Cancer. 2019 Jul 4;19:657. doi: 10.1186/s12885-019-5875-y (PMC6610844; doi:10.1186/s12885-019-5875-y)
Supplement: Supplementary file 5 — Table S1. Logistic regression analysis for factors associated with receiving RT. (DOCX 18 kb) [file 12885_2019_5875_MOESM5_ESM.docx]

**Table S1**. Logistic regression analysis for factors associated with receiving RT.

|  | Number of cases | | Chi square test | Logistic analysis | |
| --- | --- | --- | --- | --- | --- |
|  | with RT | without RT | *P* value | Odds ratio (95% CI) | *P* value |
| **Age** |  |  | 0.207 |  |  |
| <30 | 31 | 146 |  | Reference |  |
| 30-49 | 116 | 456 |  | 1.08 (0.67-1.74) | 0.748 |
| 50-69 | 237 | 857 |  | 1.22 (0.76-1.94) | 0.410 |
| 70- | 232 | 751 |  | 1.3 (0.81-2.1) | 0.276 |
| **Sex** |  |  | 0.705 |  |  |
| Male | 347 | 1226 |  | Reference |  |
| Female | 269 | 984 |  | 1.11 (0.92-1.35) | 0.281 |
| **Site** |  |  | 0.005 |  |  |
| Lower extremity | 376 | 1495 |  | Reference |  |
| Upper extremity | 128 | 353 |  | 1.87 (1.45-2.41) | <0.001 |
| Trunk | 112 | 362 |  | 1.19 (0.91-1.57) | 0.199 |
| **Depth** |  |  | <0.001 |  |  |
| Superficial | 95 | 687 |  | Reference |  |
| Deep | 521 | 1523 |  | 1.88 (1.45-2.45) | <0.001 |
| **Size** |  |  | <0.001 |  |  |
| 5cm< | 125 | 768 |  | Reference |  |
| 5cm≤, <10cm | 252 | 923 |  | 1.37 (1.06-1.77) | 0.017 |
| 10cm≤ | 239 | 519 |  | 2.08 (1.57-2.76) | <0.001 |
| **Histological diagnosis** |  |  | 0.005 |  |  |
| MLS | 85 | 264 |  | Reference |  |
| LMS | 45 | 235 |  | 0.43 (0.27-0.67) | <0.001 |
| DDLS | 59 | 150 |  | 0.56 (0.36-0.88) | 0.012 |
| MFS | 30 | 100 |  | 0.59 (0.35-0.99) | 0.045 |
| MPNST | 79 | 268 |  | 0.71 (0.48-1.06) | 0.097 |
| SySa | 34 | 136 |  | 0.55 (0.34-0.91) | 0.020 |
| UPS | 180 | 598 |  | 0.61 (0.43-0.86) | 0.005 |
| Angiosarcoma | 8 | 13 |  | 1.67 (0.63-4.42) | 0.306 |
| PLS | 18 | 60 |  | 0.60 (0.32-1.12) | 0.108 |
| Others | 78 | 386 |  | 0.49 (0.34-0.73) | <0.001 |
| **Histological grade** |  |  | <0.001 |  |  |
| Low | 63 | 483 |  | Reference |  |
| High | 553 | 1727 |  | 2.50 (1.83-3.41) | <0.001 |
| **Nodal metastasis** |  |  | <0.001 |  |  |
| Negative | 595 | 2186 |  | Reference |  |
| Positive | 21 | 24 |  | 3.72 (1.94-7.14) | <0.001 |
| **Surgical margin** |  |  | <0.001 |  |  |
| Negative | 519 | 2154 |  | Reference |  |
| Positive | 97 | 56 |  | 6.56 (4.57-9.42) | <0.001 |

RT: radiotherapy; CI: confidence interval; MLS: myxoid liposarcoma; LMS: leiomyosarcoma; DDLS: dedifferentiated liposarcoma; MPNST: malignant peripheral nerve sheath tumor; MFS: myxofibrosarcoma; SySa: synovial sarcoma; UPS: undifferentiated pleomorphic sarcoma; PLS: pleomorphic liposarcoma.
